# Efficacy of the Digital Therapeutic *sinCephalea* in the prophylaxis of migraine in patients with episodic migraine: study protocol for a digital, randomized, open-label, standard treatment controlled trial

**DOI:** 10.1186/s13063-022-06933-2

**Published:** 2022-12-12

**Authors:** Torsten Schröder, Hanna C. B. Brudermann, Gianna Kühn, Christian Sina, Diamant Thaçi, Matthias Nitschke, Inke R. König

**Affiliations:** 1 Institute of Nutritional Medicine, University Hospital of Schleswig-Holstein, Campus Lübeck & University of Lübeck, Ratzeburger Allee 160, 23538 Lübeck, Germany; 2 Perfood GmbH, Am Spargelhof 2, Lübeck, Germany; 3 Institute of Medical Biometry and Statistics, University of Lübeck, University Medical Center Schleswig-Holstein, Campus Lübeck, Ratzeburger Allee 160, 23538 Lübeck, Germany; 4 Medical Department 1, Section of Nutritional Medicine University Hospital of Schleswig-Holstein, Campus Lübeck & University of Lübeck, Ratzeburger Allee 160, 23538 Lübeck, Germany; 5 Institute and Comprehensive Center for Inflammation Medicine, University of Lübeck, Ratzeburger Allee 160, 23538 Lübeck, Germany; 6 Department of Neurology, University of Lübeck, Ratzeburger Allee 160, 23538 Lübeck, Germany

**Keywords:** Digital therapeutic, DTx, Migraine prophylaxis, sinCephalea, Digital nutrition program, RCT, Randomized controlled trial, Study protocol, Decentralized study

## Abstract

**Background:**

The German government implemented the Digital Healthcare Act in order to bring Digital Therapeutics into standard medical care. This is one of the first regulatory pathways to reimbursement for Digital Therapeutics (DTx). The Digital Therapeutic sinCephalea is intended to act as a prophylactic treatment of migraine by reducing the migraine days. For this, sinCephalea determines personalized nutritional recommendations using continuous glucose monitoring (CGM) data and enables the patients to follow a personalized low-glycemic nutrition. Migraine is a headache disorder with the highest socioeconomic burden. Emerging evidence shows that CGM-based personalized nutritional recommendations are of prophylactic use in episodic migraine. However, prospective data are yet missing to demonstrate clinical effectiveness. This study is designed to fill this gap.

**Methods:**

Patients between 18 and 65 years of age with proven migraine and a minimal disease severity of 3 migraine days per month are included. After a 4-week baseline phase as a pre-study, patients are randomized to the DTx intervention or a waiting-list control. The objective of the study is to show differences between the intervention and control groups regarding the change of migraine symptoms and of effects of migraine on daily life.

**Discussion:**

To our knowledge, this is the first systematic clinical trial with a fully digital program to enable patients with migraine to follow a personalized low-glycemic nutrition in order to reduce their number of migraine days and the migraine-induced impact on daily life. Designing a clinical study using a digital intervention includes some obstacles, which are addressed in this study approach.

**Trial registration:**

German Registry of Clinical Studies (Deutsches Register Klinischer Studien) DRKS-ID DRKS00024657. Registered on March 8, 2021.

## Background

Germany is at the forefront of digital health innovation. The Act to Improve Healthcare Provision through Digitalization and Innovation (Digital Healthcare Act – DVG) was approved on November 7, 2019, by the *Bundestag* and adopted on November 29, 2019, by the *Bundesrat* [1]. One central innovation is a regulatory framework to bring Digital Therapeutics (DTx) into standard medical care. DTx are “patient-facing software applications that help patients treat, prevent, or manage a disease and that have a proven clinical benefit” [2].

In Germany, physicians are now able to prescribe DTx to their patients. Correspondingly, statutory health insurance, covering approximately 90% of all Germans, covers the costs for DTx. After the DTx has been tested for safety, functionality, quality, data security, and data protection by the Federal Institute for Drugs and Medical Devices (BfArM), a DTx will receive the status of a DiGA (digital health application for German *digitale Gesundheitsanwendung*) and can be considered the equivalent of a prescription DTx [3].

One major step in the approval process is that the manufacturer must deliver confirmatory clinical data for permanent DiGA approval. The experience of the first months with this new legislation covering DTx is that the current gold standard to bring confirmatory data about the clinical effectiveness can be considered to be a randomized controlled trial with an open-label control group organized as the waiting control.

This study protocol has the purpose to show a prophylactic effect of the use of the DTx sinCephalea on migraine [4]. sinCephalea is the first of its kind DTx, which determines personalized nutritional recommendations using continuous glucose monitoring data and enables the patients to follow a personalized low-glycemic nutrition.

Migraine is a major contributor to disability throughout the world [5–8]. Migraine patients usually report that their performance and daily lives are impaired by headaches, and many migraine patients report a loss of productivity due to absenteeism and presenteeism at work [8, 9]. A study found that a total of one-third of patients were not receiving guideline-based therapy, i.e., did not have a prophylactically effective medication, although indicated [10]. Other studies suggest that 80% of patients with episodic migraine discontinue prophylactic medication within the first year [11, 12]. The reason may be that many of these drugs can lead to numerous adverse events such as dizziness, diarrhea, fatigue, weight gain, or erectile dysfunction [11, 12]. These are among the reasons why many patients are interested in non-pharmacological treatment strategies, such as nutrition. However, specific dietary interventions are not part of the current standard of care, although more than two-thirds of all migraine patients report their diet as a trigger on migraine activity, such as prolonged periods of fasting, alcohol, or distinct food [13]. Diets that reduce and stabilize blood glucose levels achieve improvement in migraine symptoms. A 3-month carbohydrate-modified diet reduced migraine severity, and the authors concluded that a low-glycemic diet is an effective and reliable method of migraine prophylaxis without risks for adverse drug effects [14]. It has now been repeatedly shown that postprandial blood glucose metabolism is regulated differently between individuals. Low-glycemic dietary recommendations should therefore be personalized based on individual blood glucose metabolism [15–18]. Personalization of dietary recommendations also leads to significantly higher treatment adherence [19]. The DTx sinCephalea is designed to fulfill this medical need as it was first demonstrated by own proof-of-concept data showing that an individualized low-glycemic diet based on continuous glucose measurement could be a promising approach for a diet-based, non-pharmacological migraine prophylaxis [20]. This study design aims to deliver prospective clinical data demonstrating clinical effectiveness.

## Methods/design

### Population

Patients (m/f/d) between 18 and 65 years of age with migraine according to ICD-10 codes G43.0 and G43.1 or the International Classification of Headache Disorders (ICHD-3) for migraine without aura (diagnosis 1.1 of ICHD-3) and for migraine with aura (diagnosis 1.2 of ICHD-3; all subtypes) constitute the study population. A minimal disease severity of 3 migraine days per month and study protocol compliance during the baseline phase is required to qualify for randomization.

### Study purpose

The purpose of this study is to show superiority in the form of a prophylactic effect of the use of the DTx sinCephalea on migraine. Primarily, this would be indicated by a reduction in the frequency of migraine. Further relevant parameters are a reduction in disease-associated limitations in everyday life, quality of life, and acute medication.

### Study objectives and hypotheses

The objective of the study is to show differences between intervention (IV) and control (CO) group regarding the change of migraine symptoms and of effects of migraine on daily life.

Specifically, the primary objective is to show a difference in the change in the number of days with migraine headaches in the past 4 weeks between baseline and after 12 weeks of intervention or randomization.

Secondary objectives are to show differences:

- Between adhering patients in IV and CO regarding change in the number of days with migraine headaches in 4 weeks after 12 weeks of intervention (IV) or randomization (CO) compared with baseline;
- Between IV and CO regarding response after 12 weeks of intervention (IV) or randomization (CO) as indicated by a relative reduction of the number of migraine days in 4 weeks by 30% compared with baseline;
- Between IV and CO regarding change in the limitations in daily life (headache impact) after 12 weeks of intervention (IV) or randomization (CO) compared with baseline;
- Between IV and CO regarding change in the headache-caused disability (migraine disability) after 12 weeks of intervention (IV) or randomization (CO) compared with baseline;
- Between IV and CO regarding change in the quality of life after 12 weeks of intervention (IV) or randomization (CO) compared with baseline; and
- Between IV and CO regarding change in the days with acute migraine-specific medication after 12 weeks of intervention (IV) or randomization (CO) compared with baseline.

The primary hypothesis of the study is that there is a difference in intraindividual changes in the number of days with migraine headaches in the last 4 weeks between baseline and after 12 weeks of intervention (IV) or randomization (CO).

Secondary hypotheses are that there are respective intraindividual changes with respect to further aspects of migraine severity and symptoms and migraine-related limitations of everyday life.

### Study design

The study is a randomized, open-label, intervention study controlled against the standard of care. The study is planned as a monocentric study, so that every study participant has the same examination conditions and no standardization across several study centers is necessary. However, all visits are organized digitally using telemedicine techniques qualifying the study as “decentralized.”

The study design follows the recommendations of the International Headache Society for conducting trials of medications for prophylaxis in patients with episodic migraine and the recommendations for the use of health technologies in the treatment of migraine [21, 22].

After qualification in a 4-week pre-study (baseline phase) for the main study, the study participants will be randomized 1:1 to IV and CO. The intervention is the application of the DTx sinCephalea, which determines the personalized nutritional recommendation using data from a 10-day long continuous glucose monitoring (CGM) test phase followed by a 12-week nutritional intervention.

The control group represents the non-application of the DTx according to the standard of care. Like in the intervention group, the study endpoints are also collected electronically via the smartphone. In the sense of a waiting control group, the control group is offered the use of the DTx after completion of the study outside of the protocol.

The current standard of care in Germany is that patients with migraine do not have access to a prescription non-drug treatment option. The established treatment of migraine shall be continued unchanged (standard treatment, “standard of care”).

**Fig. 1:**
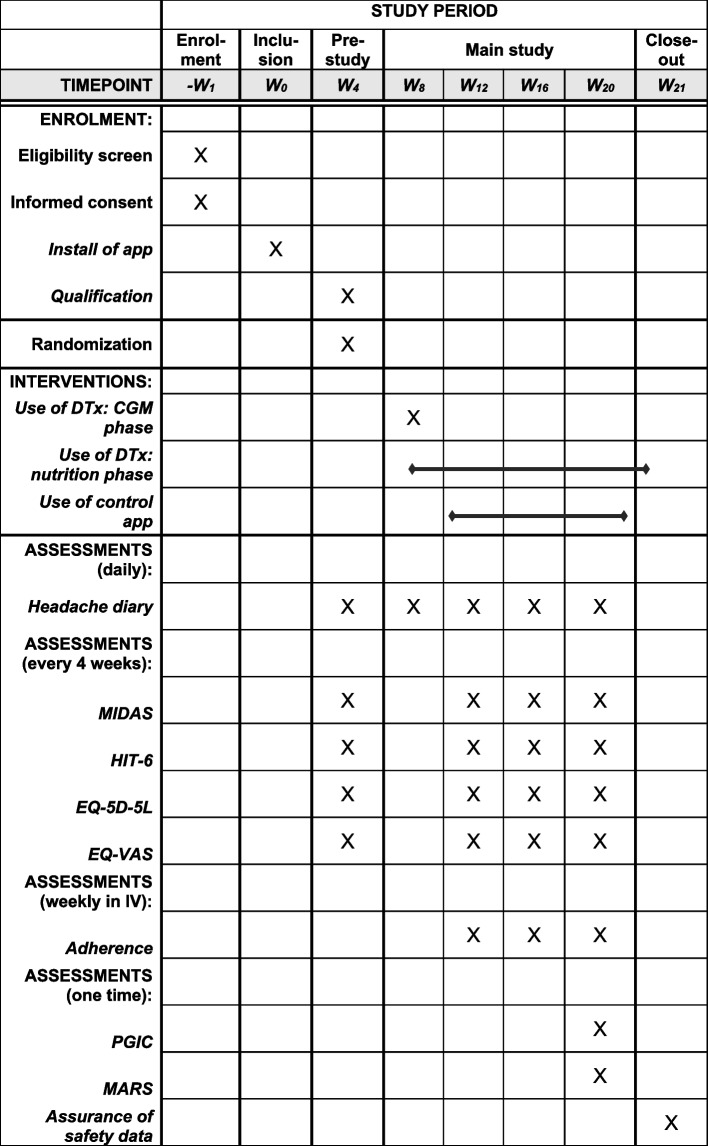
Study design in accordance to the SPIRIT guidelines

The study design is also displayed in Figure 1 using the SPIRIT reporting guidelines [23].

### Recruitment of participants

The study is organized as a digital study. All participants will be recruited from anywhere in Germany. The recruitment is done with the help of Perfood GmbH using its established database of individuals who have indicated that they suffer from migraine and are interested in participating in a clinical trial. Furthermore, study information material will be distributed to physicians, who regularly treat migraine patients, and interested subjects via a specific homepage, newsletters, as well as social media campaign channels (e.g., Facebook, Instagram). Interested patients and physicians will be informed about the inclusion and exclusion criteria as well as the study procedure via the website of Perfood GmbH and pre-screened by means of an online survey in compliance with the inclusion and exclusion criteria. Recruitment processes will be undertaken anonymously and follow all rules of the EU General Data Protection Regulations (GDPR). Pre-screened individuals will make a digital appointment at the study site for further information and the inclusion process will be supervised by the PI. A model consent form with planning data is an Appendix to this paper.

### Registration

During the inclusion visit, the patient is informed about the course of the study, and the following inclusion and exclusion criteria for the pre-study (baseline phase) are checked.

### Inclusion criteria

- Migraine according to Chapter 1 of ICHD-3
- Average of 3 or more migraine days per 4 weeks
- Ability to distinguish between migraine and other headaches
- Minimum 18 years of age and maximum 65 years (at the time of inclusion).
- Onset of migraine before the age of 50 and existence for at least 12 months
- Use of an Android (from version 5.1) or iOS smartphone (from version 13.0) (use of the sinCephalea app required)
- Sufficient knowledge of German to understand the study materials
- Sufficient intellectual capacity to consent and participate
- Ability and willingness to provide consent
- Written consent

### Exclusion criteria

- Use of another Perfood GmbH product with a continuous glucose analysis within the last 24 months.
- Pregnancy, current desire to have children, breastfeeding
- Evidence of another type of headache
  - Chronic migraine according to chapter 1 of ICHD-3
  - Primary headache according to ICHD-3 chapters 3 and 4 (trigeminal autonomic headache disorders; other primary headache disorders)
  - Medication overuse headache
  - Tension-type headache (ICHD-3, Chapter 2) is not an exclusion criterion provided that this headache has accounted for less than 50% of headache days per 4 weeks within the past 3 months and there is an indication for prophylaxis because of migraine symptoms.
- Complicated migraine attacks with debilitating (e.g., hemiplegic) and/or, long-lasting auras, following a migrainous cerebral infarction
- Change in prophylactic medication or use of an alternative migraine app (other than for headache recording only) within the past 12 weeks
- Drug prophylaxis of migraine with more than one preparation (including medication for other illnesses that are being treated with medication, which can also be used to prevent migraine with medication, such as for arterial hypertension treated with beta-blockers, ACE inhibitors or AT1 antagonists, and explicitly the use of amitriptyline, valproate, topiramate, or flunarizine)
- Non-drug treatment of migraine with acupuncture
- Diagnosis of a malignant disease within the last 3 years or during ongoing therapy
- Insulin-treated diabetes mellitus
- Psychiatric illness (other than stably treated depression) that requires drug therapy or has required inpatient therapy within the past 12 weeks
- Eating disorder (binge-eating, anorexia nervosa, bulimia)
- Chronic pain syndrome with a need for pain medication (ICD-10 F45.4)
- Alcohol or substance abuse
- Simultaneous participation in another clinical trial

After signing the declaration of informed consent, the patient is registered in the pre-study, which is documented at the study site using clinical reporting forms (CRFs), and every patient is assigned an individual identification number as pseudonym. The sponsor only has access to the pseudonymized data and only receives pseudonymized data on the patients.

### Baseline assessment (pre-study)

After inclusion in the pre-study, there is a 4-week baseline phase for the prospective recording of the disease severity and for securing the inclusion/exclusion criteria as well as compliance (measured by the frequency of completion of the questionnaires required for the study and the headache diary for measuring the relevant endpoints). This ensures suitability according to the inclusion and exclusion criteria as well as sufficient cooperation for the valid collection of the endpoint-relevant patient-reported outcomes. This approach follows the recommendations of the International Headache Society for conducting studies on prophylactic treatments for episodic migraine [21, 22].

For participation at randomization and inclusion in the main study, the following inclusion criteria apply additionally:

- Complete answering of all questionnaires of the baseline phase (all questions)
- Use of headache diary on at least 80% of days in baseline phase (at least 22 of 28 days, daily questioning whether symptoms were present)
- At least 3 reported migraine days according to inclusion criteria during the baseline phase (4 weeks).

### Randomization

Stratified permuted block randomization is used with stratification by gender and number of migraine days reported over the past 4 weeks (<8 days vs. ≥8 days). The block lengths are fixed and documented at the Institute of Medical Biometry and Statistics, University of Lübeck (IMBS), but are not available to those who enroll participants or assign interventions. MersenneTwister is used as a random number generator based on a real random seed from www.random.org. For the generation of the allocation sequence, RITA (version 1.50) is used.

A 1:1 randomization will be carried out. The randomization takes place centrally at the IMBS. At the beginning of the study, all future participants are randomized at once (list randomization). During the study, after enrolment of a single participant, a randomization form is filled in by the study center and sent (via fax or email) to the IMBS. This form includes the study ID, information on the strata, inclusion and exclusion criteria, name of the investigator, date and signature. At the IMBS, the validity of the enrolment is checked. If correct, the participant is registered in a data base, and the next allocation in the respective strata combination is selected. The allocation is noted on the randomization form, which is then signed and sent back to the study center.

Given the complex nature of the intervention, a blinding of the patients is not possible. The primary and secondary endpoints are based on patients’ assessment and therefore are not blinded.

### Main study

After randomization, the main study comparing IV with CO begins. The use of the DTx represents the treatment in the IV phase after randomization. The DTx determines the personalized nutritional recommendation using data from a 10-day long CGM test phase followed by a 12-week nutritional intervention. The structure of the intervention phase is the same for all patients in IV, but the implemented nutritional recommendations are personalized.

A test kit and the use of one of the validated tissue glucose sensors (Dexcom G6 sensor and Abbott FreeStyle Libre sensors 1 and 2) are required for the IV. Immediately after randomization, the study site sends the test kits to the patient. After receiving the test kit, the individual test phase begins. For this purpose, the tissue glucose sensor is first applied according to the manufacturer’s instructions and remains there for at least 10 days. At the same time, food intake and other events such as sleep, everyday movement, and physical activity are recorded via the app. The state of health, stress, and migraine-specific symptoms are also logged via the app. The participants continue their usual eating habits and test the meals they like and eat frequently.

All data will be computed by Perfood GmbH in accordance with the sponsor’s internal SOPs, and the personalized nutrition recommendations are sent to the individual patients via their app. Immediately after receipt, consultations with nutrition experts are conducted voluntarily to ensure that the patient correctly understands all recommendations and are able to adjust his diet accordingly. Recommendations are also well explained in the app, ensuring understanding of the recommendations even if nutritional consulting is not realized. The intervention phase, in which the personalized nutritional recommendations are implemented, lasts 12 weeks until the endpoint is recorded. It thus represents the actual migraine prophylaxis by adhering to the personalized low-glycemic diet.

The CO is the non-application of the DTx while continuing the standard treatment. The study participants are provided with an app through which only the study-relevant questionnaires can be used. This ensures that there is no difference between the groups regarding the mode of assessment of the patient-reported outcomes. After the follow-up after 12 weeks and the individual end of the study, the DTx can be used.

Known or foreseeable factors that may affect the outcome of the clinical examination or interpretation of the results have been considered as part of the inclusion and exclusion criteria. Beyond these factors, the use of acute medications for migraine attacks (analgesics, triptans) may be continued as needed. Patients not previously treated with any prophylactic medication, as well as patients already receiving such medication, may be included. During the intervention, this should be continued unchanged.

### Sample size

The sample size was calculated with the aim to show a difference in the change in the number of days with migraine symptoms in the past 4 weeks at follow-up 12 compared with baseline.

To estimate the possible effect in the control group, eight studies were identified in which migraine patients in a control group received standard care or were placed in a waiting group [24–31]. These control patients were all aware of being in a control group, which is comparable to the planned study situation. Mean changes in migraine days with corresponding standard deviations and sample sizes were extracted from these publications and meta-analyzed using a random effects model. This yielded a pooled estimate of the mean change of 1.14 days.

The sample size was then calculated based on the following assumptions:

- Patients in the control group will experience a reduction similar to the reduction observed in the control groups in the literature, which was a reduction by 1.14 days on average.
- Patients in the intervention group will experience a reduction of at least 50% on average, which is recommended to be the minimally relevant effect [21]. Assuming that, at baseline, they will report a mean of 4.26 days as in our pre-study (internal data), this reduction corresponds to a mean change by 2.13 days.
- The drop-out rate is conservatively estimated to be 20% in every group. Although for the analysis of the primary endpoint, missing values will be imputed by multiple imputation, we assume a reduction of 0 days in patients with missing data for a conservative estimation of the sample size.
- In the intervention group, the average change is therefore estimated to be (0 days) × 20% + (2.13 days) × 80% = 1.704 days. In the control group, the average effect is therefore estimated to be (0 days) × 20% + (1.14 days) × 80% = 0.912 days.
- Based on values in the pre-study, the standard deviation of the change is set to 3.19 days.
- Significance level and power are set to α=0.05 (two-sided) and 1-β=0.8.
- To detect this effect using a two-group *t*-test requires that 256 patients per group are randomized (nQuery 4.0) [32].

### Data assessment

Data are recorded as patient-reported outcomes with validated questionnaires as recommended by the International Headache Society [21, 22]. The questionnaires are answered in digital form via the app (eDiary). These questionnaires include the following:

- Participants are surveyed daily using an electronic headache diary. This defines the migraine days and records the benefit of acute medication.
- The MIDAS (Migraine Disability Assessment) questionnaire is answered every 4 weeks to assess headache-related impairment [33–35].
- The HIT-6 (Headache Impact Test 6-item) is answered every 4 weeks to record impairment in daily life [36, 37].
- The EQ-5D-5L to assess the quality of life is answered every 4 weeks. The EQ-VAS is used for patient assessment of health status [38].
- During the intervention, questions about adherence to dietary recommendations will be asked every 7 days.
- The Patient Global Impression of Change (PGIC) is applied at the end of the intervention for patient assessment of change in symptoms [39].
- Medication Adherence Report Scale (MARS) is used at the end of intervention and control to determine adherence to therapy with existing prophylactic drug therapy [40, 41].

### Data management

Our app allows for various types of input, including all above-mentioned validated migraine questionnaires, a daily headache diary, and a digital nutrition diary. This data is stored in a local, encrypted database on the user’s device and is securely synchronized to Perfood servers, where it is stored encrypted, backed up regularly, and accessible only by specifically trained people following Perfood’s SOPs. All servers reside in Germany fulfilling high standards regarding security and stability required by law for DTx in Germany.

CGM data are collected on the reading devices from the CGM manufacturer and then are transferred by the users to enter our servers. CRF data are independently digitalized twice in order to avoid errors.

All data is checked for consistency and data value ranges. Questionnaires can only be submitted if all mandatory fields are filled out. All data processing is tested using unit and system tests. The final database is archived in a way to ensure a minimum of 10 years.

### Endpoints

The primary efficacy endpoint of this study is the change in the number of days with migraine symptoms in the past 4 weeks at 12 weeks after intervention in IV or after randomization in CO compared with baseline. For this, days with migraine headache are counted from the electronic headache diary of the past 4 weeks at baseline and at follow-up 12.

Secondary efficacy endpoints are:

1. Change in the number of days with migraine symptoms at follow-up 12 compared with baseline after exclusion of non-adhering patients in IV
2. Response as indicated by a relative reduction of the number of migraine days assessed by the eDiary in 4 weeks at follow-up 12 by 30% compared with baseline
3. Change in the limitations in daily life (assessed by HIT-6) at follow-up 12 compared with baseline
4. Change in the headache-caused disability (migraine disability, assessed by MIDAS) at follow-up 12 compared with baseline
5. Change in the quality of life (assessed by EQ-5D-5L) at follow-up 12 compared with baseline
6. Change in the days with acute migraine-specific medication (assessed by eDiary) at follow-up 12 compared with baseline

Exploratory endpoints will include the change in the number of days with migraine symptoms, the response, the change in headache-caused disability, and in acute migraine-specific medication defined above at earlier time points, i.e., at follow-ups 4 and 8.

Furthermore, at all time points, the following endpoints compared with baseline will be assessed:

- Response as indicated by a relative reduction of the number of migraine days in 4 weeks by 50% compared with baseline (eDiary),
- Change in number of days with non-migraine headaches of moderate or severe intensity in the past 4 weeks (eDiary),
- Change in number of migraine attacks in the past 4 weeks (eDiary),
- Change in maximal headache pain intensity in the past 4 weeks (eDiary),
- Change in cumulative length in hours of migraine attacks in the past 4 weeks (eDiary),
- Change in cumulative length in hours of non-migraine headache in the past 4 weeks (eDiary),
- Change in self-assessment on health state (EQ-VAS),
- Change in missed days at work, school, or equivalents in the past 4 weeks (MIDAS),
- Change in productivity at work, school, or equivalents in the past 4 weeks (MIDAS),
- Adherence to existing medication (MARS),
- Assessment on impression of change in symptoms (Patient Global Impression of Change, only follow-up 12), and
- Change in number of days without headaches in 4 weeks (eDiary)

Adherence endpoints will be assessed to evaluate how strictly the patients adhered to the recommendations at the end of every week of intervention, totaling 12 time points. At each of these time points, the number of days a specific meal (breakfast, lunch, dinner, snack, beverage) was consumed is assessed as well as the number of days the recommendations for the specific meal were largely followed. From this, the total number of meals per week and the percentage of meals with followed recommendations are computed for every patient. From this, a patient will be defined to be non-adherent if the percentage of meals with followed recommendations is < 50%.

As safety endpoints, all adverse events (AEs) and serious adverse events (SAEs) as well as adverse device effects (ADEs) will be monitored.

### Quality control and quality assurance

The study-independent quality control and assurance is ensured by monitoring of the Center of Clinical Studies (ZKS, Zentrum für Klinische Studien) Lübeck. The risk-based monitoring is performed according to ISO 14155:2020 as well as own SOPs.

### Statistical analysis

For all analyses, all patients will be considered who were successfully randomized and gave their informed consent.

The primary endpoint and other efficacy endpoints will be evaluated in the full analysis (FA) set in which patients, in whom it becomes apparent after randomization that they have either (1) headaches other than migraine or tension-type, if the latter led to more than 50% of the headache days per month; or (2) 14 or more days with migraine headaches per month, are excluded. As a sensitivity analysis, all efficacy endpoints will be additionally evaluated in the intention to treat (ITT) and per protocol (PP) populations. Here, PP additionally excludes patients in whom the intervention was not started, who did not adhere to the recommendations as defined above, changed prophylactic treatment with change of the dose, a switch to another medication, or addition of another medication, or entered no data after the first 4 weeks of IV/CO. Safety endpoints will be evaluated in the safety analysis (SA) set that includes all randomized patients, but patients in IV in whom the intervention was not started or who did not adhere to the recommendations are analyzed as CO participants.

All variables at all time points and all defined primary, secondary and exploratory endpoints (i.e., changes from baseline to follow-up visits) will be described according to their type (measurement, normal, log-normal, ordinal, proportion) using standard metrics in all analysis sets. The disposition of patients will be described by a CONSORT flow chart.

For the primary objective regarding the primary efficacy endpoint, the primary hypothesis will be tested using a linear model in the FA analysis set. With *y*_*i*_ denoting the endpoint “change in the number of days with migraine symptoms at 12 weeks” in patient *i*, the linear model is defined as follows:

$${y}_i={\beta}_0+{\beta}_1{x}_i^{\textrm{Treat}}+{\beta}_2{x}_i^{\textrm{Sex}}+{\beta}_3{x}_i^{\textrm{Baseline}}+{\varepsilon}_i,$$

where

$${x}_i^{\textrm{Treat}}=\left\{\begin{array}{cc}\ 0,&\ \textrm{if}\ \textrm{CO},\\ {}\ 1,& \textrm{if}\ \textrm{IV}\end{array}\right.$$, $${x}_i^{\textrm{Sex}}=\left\{\begin{array}{cc}\ 0,&\ \textrm{if}\ \textrm{male},\\ {}\ 1,& \textrm{if}\ \textrm{female}\end{array}\right.$$, and ${x}_i^{\textrm{Baseline}}$ is given by the number of days with migraine symptoms at baseline.

We test the hypotheses H0: *β*_1_ = 0 vs. H1: *β*_1_ ≠ 0with a linear model assuming variance homogeneity using a two-sided Wald test at significance level *α* = 0.05. The corresponding 95% Wald confidence interval will be estimated.

A sequential testing procedure will be used to maintain a family-wise type I error of 0.05 for primary and secondary endpoints. If the primary endpoint is statistically significant at *α* = 0.05, the secondary efficacy endpoints will be tested. Hence, positive results on secondary endpoints can be interpreted inferentially only if a treatment effect is shown on the primary endpoint (gate-keeping).

As a further gate-keeping test, the first secondary endpoint (change in the number of days with migraine symptoms at follow-up 12 compared with baseline after exclusion of non-adhering patients in IV) is tested first at *α* = 0.05. If this is significant, the other five secondary endpoints will be tested with significance levels adjusted according to Bonferroni-Holm [42]. If any null hypothesis is rejected, a difference between IV and CO in the respective endpoint is shown. If the null hypothesis is not rejected, it will not have been shown that there is a difference.

### Missing data

#### The endpoints

- Number of days with migraine headaches in the past 4 weeks,
- Number of days with acute migraine-specific medication in the past 4 weeks,
- Number of days with non-migraine headaches of moderate or severe intensity in the past 4 weeks,
- Number of migraine attacks in the past 4 weeks,
- Number of days without headaches in the past 4 weeks,
- Cumulative length in hours of migraine in the past 4 weeks, and
- Cumulative length in hours of non-migraine headaches in the past 4 weeks

are based on counting the number of days, attacks, or hours, respectively, from the headache eDiary. The respective endpoints will be set to missing if patients made entries for less than 80% of the days, i.e., for less than 22 out of 28 days. If patients made entries for at least 80% but less than 100% of the days, the missing number of days, attacks, or hours will be imputed by the average of the valid entries.

The primary efficacy endpoint of this study is based on the number of days with migraine symptoms in the past 4 weeks at 12 weeks after intervention in IV or after randomization in CO (FU12). At FU12, missing values might occur for several reasons. Generally, we assume that patients in CO are likely to stop diary entries for no specific reason. Missing values in IV might occur in the following scenarios:

1. Patients in IV might stop the intervention and corresponding diary entries early because of no effect. Their missing values should therefore be comparable to the values in CO, and this scenario hence assumes that values are missing not at random (MNAR).
2. Patients in IV might stop the intervention and corresponding diary entries early for other reasons not related to the outcome. Alternatively, patients in IV might only stop diary entries but keep the same level of adherence. Thus, missing values should be comparable to the observed values in IV, and this scenario assumes that values are missing at random (MAR) or missing completely at random (MCAR).

For the primary endpoint analysis, we assume scenario 1. Missing values are multiply imputed via construction of a joint distribution of the patients’ observed and missing data. This uses the jump-to-reference method as a special pattern mixture model, for which the distribution from CO is set as in reference [43]. From the generated imputation samples, *β*_1_ will be estimated using the above model in every data set. The estimates are then combined using Rubin’s rule [44]. Further details will be specified in the statistical analysis plan (SAP).

No other imputations of missing data will be performed, and all other analyses will be based on complete cases.

### Sensitivity analyses

Sensitivity analyses of the primary endpoint will include variations in the handling of missing data. While details will be specified in the SAP, these will include (a) multiple imputation under the MAR assumption as in scenario 2 [45]; (b) complete cases analysis without imputation under the MCAR assumption; (c) imputation by baseline observation carried forward based on the baseline assessment in the FA set as a conservative approach assuming that withdrawing patients will return to their baseline values of the original levels of symptoms. Furthermore, the primary endpoint will be evaluated in the ITT and PP population. Further sensitivity analyses include the analysis of the secondary endpoints in the ITT and PP populations.

## Discussion

This paper describes the design and the methodology of a randomized controlled trial on the clinical effectiveness of the DTx sinCephalea as prophylaxis of migraine. To the best of our knowledge, this is the first systematic clinical trial with a fully digital program to enable patients with migraine to follow a personalized low-glycemic nutrition in order to reduce their number of migraine days and the migraine-induced impact on daily life.

The study is designed to fulfill the regulatory requirements of the newly established Digital Healthcare Act bringing DTx into standard medical care. The clinical data will be handed to the Federal Institute for Drugs and Medical Devices (BfArM) for a thorough assessment. Confirmatory clinical data showing the superiority of the application of the DTx over the non-application of the DTx is required so that the DTx can be prescribed and will be fully reimbursed by the German statutory health insurance system.

Designing a clinical study using a DTx includes some obstacles, which must be addressed. DTx usually facilitate complex interventions with more than just one active ingredient as it would be the case in pharmacological treatments. Frequently, DTx are designed to allow lifestyle modifications, tracking of several activities, parameters, and conditions as well as using several channels for disease-specific educations. Consequently, a blinded control app is often out of the possibilities. A blinded control would require that at least the patient does not recognize whether he or she uses the intervention or the control DTx. There is the concept of a “sham-DTx” as an equivalent to a placebo control [46]; however, it has to be acknowledged that complex DTx with multiple possible ingredients potentially responsible for a clinical effect cannot be controlled well with a reductive version of the DTx [47, 48]. Consequently, the current gold standard for DTx studies is designing the control group as a wait-list control into which the patients are randomized [49–52]. This control group design is open-label by design and brings the obstacle of a missing blinding. Subsequently, it is possible that effect estimates will be biased by a “placebo effect”. This “placebo effect” might act on two levels: First, patients might be affected by the participation in the trial itself and the regular logging of symptoms, thus experiencing a reduction of symptoms. This might apply to both patient groups and thus not systematically bias effect estimates. Second, patients in the intervention group might additionally be affected by the knowledge of being in the intervention group, which might lead to an inflation of the effect when comparing the intervention group with the control group. To explore the extent of bias, in our study the adherence of patients to the recommendations will be assessed, and exploratory analyses will correlate the extent of adherence with the effects on migraine. Finally, results from this study are only applicable to patients who are able and willing to use this DTx. Comparing the baseline characteristics at inclusion visit between patients who do and do not participate in the main study will help to estimate the extent of this bias.

Another interesting aspect of working with DTx in a clinical study is that the use of a DTx brings the opportunity to include a digital outcome assessment. Digitally assessing the relevant outcomes has huge advantages: Patients can be reminded using app notifications, time stamps give information about the real timepoint of answering questionnaires, and in real-time the answers can be examined for consistency [53]. On the other hand, it has to be assured that the control group not using the intervention DTx uses a comparable manner of outcome assessment excluding a potential bias. In the current study, all participants in the control group use a modification of the intervention DTx which only allows for digitally answering the questionnaires. After completion of the study, all participants in the control group are offered to use the DTx, as part of a compensation of their effort.

A third obstacle by design is that apps and digital applications are frequently merely tested, and users may lose interest. Consequently, studies with apps and DTx usually face high rates of discontinued use. As well some participants are lost to follow up or simply drop out [54]. To address this issue, we only randomize participants who regularly used the app during the pre-study, thus indicating a long-term interest in its application.

This study is the first RCT with a completely digital nutrition program as prophylaxis of migraine and will bring valuable clinical data allowing confirmatory prove of the effectiveness of the DTx sinCephalea.

## Trial status

This trial was initiated on 17 June 2021 and is currently in the recruiting. The current CIP is V2.4 from 27 September 2021 including the statistical study planning document V05 from 23 August 2021.

## Data Availability

The final dataset will be handled to the Institute of Medical Biometry and Statistics, University of Lübeck for statistical analysis. A publication of the study data is planned in a scientific per-reviewed journal.

## Abbreviations

ADE: Adverse device effect
AE: Adverse event
BfArM: Federal Institute for Drugs and Medical Devices for German “Bundesinstitut für Arzneimittel und Medizinprodukte”
CGM: Continuous glucose monitor/monitoring (validated are the Dexcom G6 sensor and Abbott FreeStyle Libre sensors 1 and 2)
CIP: Clinical investigation plan
CO: Control group
CRFs: Clinical reporting forms
DiGA: Digital health application for German digitale Gesundheitsanwendung
DTx: Digital Therapeutics
DVG: Digital healthcare act for German Digitale-Versorgung-Gesetz
eDiary: Electronic diary
FA: Full analysis
FU12: Follow-up week 12 after intervention in IV or after randomization in CO
GDPR: EU General Data Protection Regulations
HIT-6: Headache Impact Test 6-item
ITT: Intention to treat
IV: Intervention group
GDPR: General Data Protection Regulations
MAR: Missing at random
MARS: Medication Adherence Report Scale
MCAR: Missing completely at random
MIDAS: Migraine Disability Assessment
MNAR: Missing not at random
PGIC: Patient Global Impression of Change
PI: Principal Investigator
PID: Patient identification
PP: Per protocol
SA: Safety analysis
SAE: Serious adverse event
SAP: Statistical analysis plan
SOPs: Standard operating procedures
ZKS: Zentrum für Klinische Studien Lübeck (Center of Clinical Studies)
